# The physician-nurse collaboration in truth disclosure: from nurses’ perspective

**DOI:** 10.1186/s12912-021-00557-8

**Published:** 2021-03-06

**Authors:** Qinqin Cheng, Yinglong Duan, Ying Wang, Qinghui Zhang, Yongyi Chen

**Affiliations:** 1 Hunan Cancer Hospital/The Affiliated Cancer Hospital of Xiangya School of Medicine, Central South University, Changsha, People’s Republic of China; 2 The Third Xiangya Hospital, Central South University, Changsha, People’s Republic of China; 3 Hunan University of Chinese Medicine, Changsha, People’s Republic of China

**Keywords:** Physician–nurse collaboration, Truth disclosure, Nurses’ perspective

## Abstract

**Background:**

Collaboration between physicians and nurses is critical. However, a limited number of studies have provided insights into the status of physician–nurse collaboration in truth disclosure.

**Methods:**

A cross-sectional survey was conducted using an electronic questionnaire among Chinese nurses who attended a provincial conference. The Nurse–Physician Collaboration Scale was administered to nurses to assess the collaboration in truth disclosure from their perspective. A multiple-choice question was asked to assess the perceived difficulties in truth disclosure. Descriptive statistics, univariate, and multiple stepwise regression analyses were performed to evaluate physician–nurse collaboration in truth disclosure.

**Results:**

A total of 287 nurses completed the survey, and 279 of them reported that they had carried out truth disclosures among patients. The average score for physician–nurse collaboration in truth disclosure was 3.98 ± 0.72. The majority of nurses (73.1–81%) responded positively to different dimensions of collaboration in truth disclosure. The results of multiple stepwise regression analysis showed that seniority (B = − 0.111, 95% confidence interval [CI] = − 0.167−− 0.055, *p* < 0.001) and frequency of truth disclosure (B = 0.162, 95%CI = 0.076–0.249, *p* < 0.001) were the only two factors associated with collaboration in truth disclosure between physicians and nurses. The most common barrier perceived by nurses was fear of patients’ negative emotions or their suicide attempts after truth telling.

**Conclusions:**

Most nurses responded positively to physician–nurse collaboration in truth disclosure. Various difficulties existed in the practice of truth-telling collaboration. Further studies are required to test the potential interventions to promote cooperation between nurses and physicians in truth disclosure.

## Background

All patients need to undergo the process of diagnosing, treatment, and eventually the terminal stage of certain diseases during their lifetime, and medical personnel must face the issue of disclosing diagnoses and disease prognosis. Thus, truth disclosure is an important aspect of caring for patients. According to the Law of the People’s Republic of China on Medical Practitioners, medical personnel should disclose the truth to the patients and their families, but they are obligated to pay attention so as to avoid adverse consequences of telling the truth in clinical practice [1]. Telling the truth in mainland China is not as optimistic. Under traditional cultural and medical contexts there, most physicians first inform patients’ families of the diagnosis and then follow the families’ willingness to decide whether to reveal the truth to patients [2]. As most families would like to hide the truth to protect the patients from psychological harm or burden, less than half of the patients know their exact diagnosis, especially cancer patients [1].

Research has proved that medical communication about the diagnosis or prognosis of patients can improve their understanding of the disease condition, increase their sense of autonomy, and encourage them to make their own decision making [3, 4]. However, this kind of medical communication entails more than merely informing patients of the diagnosis or prognosis. It also concerns how physicians or other health care staff can support the patient emotionally by finding a personalized way of disclosure according to individual preferences [5–7]. Many medical personnel reveal that it is quite difficult to inform patients of their diagnosis or prognosis appropriately [8, 9]. Furthermore, medical personnel disclosing a disease may develop negative emotions that would affect their work. Consequently, the issue of how to disclose the truth is more important than whether to do so [10]. Many researchers advocate that it is better for physicians and nurses to cooperate in truth telling [11, 12].

Collaboration implies interactions in which professionals work together cooperatively, with shared responsibility and interdependence [13]. Physician–nurse collaboration is a critical element of truth disclosure, which can facilitate information exchange and help them know what has been communicated to patients or families [14]. In addition, collaboration between physicians and nurses can not only reduce staff distress [15] and improve staff satisfaction [15, 16] but also improve patient outcomes [17, 18] and satisfaction [15, 19]. However, truth telling is considered to be the responsibility of physicians in most cases [12]. Nurses are often not involved in the disclosure process because most of them do not take truth telling as their duties [20]. Nurses play an integral role in diagnostic and prognostic disclosure [21]. Many patients with cancer have indicated that they also look to nurses to gain an understanding of prognosis when making difficult treatment decisions [22, 23].

These findings highlight the importance of physician–nurse collaboration in truth disclosure. However, there are little data on the degree of collaboration between physicians and nurses and the influencing factors. Thus, the present study aimed to investigate the status of physician–nurse collaboration in truth disclosure from nurses’ perspectives and explore the potential influencing factors.

## Methods

### Participants

We conducted a cross-sectional survey among nurses who attended a provincial oncology nursing conference held in Changsha, Hunan Province, in 2019. Potential participants were recruited from the nurses who participated in this conference. The inclusion criteria were (1) registered nurses and (2) voluntary participation in the study.

### Instrument

The questionnaire consisted of three parts. In the first part, there were seven items regarding participant characteristics. The second part was the questionnaire used to measure the frequency of cooperative activity and to verify the specific relationships between nurses and physicians in truth disclosure. We used the Nurse–Physician Collaboration Scale (NPCS) developed by Ushiro (2009) [24]. It was translated into Chinese by Jing Chen and adapted to expressions and semantics to reflect the Chinese culture [25]. The Chinese version of the NPCS was validated and proved to have a relatively high reliability and validity when used to evaluate the status of nurse–physician collaboration in the Chinese medical environment [25]. It had 21 items in total and could be divided into three dimensions: sharing of patient information, joint participation in the cure/care decision-making process, and cooperativeness. In this study, we changed the words “care or decision-making process” to “truth disclosure” to lead the participants to complete the questionnaire from the perspective of truth disclosure. However, these modifications did not change the essence of the questions. All items were rated on a 5-point Likert scale from “never” to “always,” scoring from “1” to “5”. A higher score indicates better cooperation between nurses and physicians in truth telling. To categorize the response, we defined a mean answer of > 3 on the questionnaire items as a positive response [26]. Cronbach’s α coefficient was 0.970 in the present study, indicating sound reliability. In the third part, a multiple-choice question was provided to assess participants’ perceived difficulties in truth telling.

### Survey method and data collection

The survey was conducted using an online survey platform (Wen Juan Xing®, www.wjx.cn). The conference attendees were invited to participate in the investigation on a voluntary basis. Before completing the questionnaire, the participants were informed of this survey in the introduction to the survey questionnaire, which involved the survey purpose, contents, benefits or risks, length of time to complete the survey, and gratefulness for their participation. The survey took about 10 min to complete, and no financial incentives were provided. Each participant could only complete the survey once, as the QR code linking prevented duplicate filling out. All information, including WeChat ID and IP address, was confidential and only used for this research.

### Analysis

All data were analyzed using the SPSS Statistics software (version 18.0; IBM, Armonk, NY, USA). Descriptive statistics were used to describe the characteristics of the nurses and their responses to the questionnaire. Continuous variables (participants’ age, clinical experience [year], and scores of different dimensions of nurse–physician cooperation in truth telling) were presented as means and standard deviation (SD), while categorical variables (participants’ age [grouping], sex, education, hospital type, clinical experience [grouping], seniority, and frequency of truth disclosure) were expressed as numbers and percentages. The *t*-test and analysis of variance were used to evaluate and compare the differences between the groups. Multiple stepwise regression analysis was used to determine the factors related to collaboration. A *p*-value of <.05 was considered statistically significant.

## Results

### Characteristics of the participants

In total, we received 294 questionnaires, of which 287 (97.6%) were valid. As our study focused on collaboration in patients’ truth telling, we excluded respondents who never implemented truth telling. Finally, 279 participants (97.2%) were included in our analyses. The characteristics of the study participants are listed in Table 1. The mean age of the participants was 34.76 (7.51), and the vast majority (97.8%) were female. Most of them (73.5%) had a bachelor’s degree. The participants worked predominately in tertiary care hospitals (81.0%), and the average time of clinical experience was 14.17 (8.53) years. More than one-third of the participants had an intermediate title. Less than half of them said that they sometimes told the truth to patients.

**Table 1:** Characteristic of participants (*n* = 279)

Variables	*n*	%
Age		
≤ 25	36	12.9
26–35	115	41.2
36–45	101	36.2
≥ 46	27	9.7
Sex		
Male	6	2.2
Female	273	97.8
Education		
Associate’s degree	63	22.6
Bachelor’s degree	205	73.5
Master’s degree	11	3.9
Hospital type^*^		
Tertiary hospital	226	81.0
Secondary hospital	53	19.0
Clinical experience (year)		
≤ 5	46	16.5
6–10	67	24.0
11–15	53	19.0
16–20	44	15.8
≥ 21	69	24.7
Seniority (clinical)^§^		
Primary title	85	30.5
Intermediate title	112	40.1
Senior title	82	29.4
Frequency of truth telling		
Seldom	43	15.4
Sometimes	116	41.6
Often	77	27.6
Always	43	15.4

^*^ According to <General hospital classification management standards (trial) > issued by Ministry of Health of the People’s Republic of China in 1989, the hospitals were divided into three levels (first level hospital, secondary hospital, and tertiary hospital) in mainland China depending on the tasks and functions. Usually, tertiary hospital offers a wide range of health services and undertakes more complex medical tasks compared to secondary hospital [27]

^§^Seniority (clinical) is a reflection of professional title depending on education and working experience. The nurses who meet the requirements of education level and working experience can apply for the certification examination of certain professional title. Those who can pass the examination can get a certificate of a certain professional title (primary, intermediate, or senior)

### Nurse–physician collaboration in truth telling

Table 2 shows means (SD), range of average score, and percentage of positive responses of each dimension. The average score of NPCS was 3.98 ± 0.72. The participants got a mean score from 3.94 to 4.06 in different dimensions of nurse–physician cooperation in truth telling. Overall, 77.4% of the participants gave positive responses (score 4–5).

**Table 2:** Nurse–Physician Collaboration Scale, mean scores, positive response percentages (*n* = 279)

Variable	Mean (SD)	Range	Positive percentage
Sharing of patient information	4.01 (0.72)	1–5	81.0%
Joint participation in truth telling	3.94 (0.82)	1–5	73.1%
Cooperativeness	4.06 (0.78)	1–5	78.5%
Total mean score	3.98 (0.72)	1–5	77.4%

### Factors related to physician–nurse collaboration in truth telling

Univariate analysis (Table 3) showed that nurses’ scores did not vary significantly by sex, education, or hospital type. However, nurses’ age, clinical experience, seniority, and the frequency of truth disclosure showed significant relationships with their collaboration with physicians in truth disclosure (*p* < 0.05). Multiple linear regression analysis was carried out when the score of nurse–physician collaboration was set as the dependent variable and the factors (age, clinical experience, seniority, and frequency of truth disclosure) that had significant relationships with collaboration in univariate analysis were independent variables. The results showed that seniority and frequency of truth disclosure were the only two factors associated with cooperation in truth telling among nurses and physicians (Table 4). Nurses who had lower levels of seniority and carried out more truth disclosures had better cooperation with physicians in telling patients’ truth.

**Table 3:** Results of univariate analysis (*n* = 279)

Variables	Mean (SD)	T/F value	*p*
Age		7.056	< 0.001
≤ 25	4.17 (0.66)		
26–35	4.15 (0.71)		
36–45	3.74 (0.72)		
≥ 46	3.95 (0.53)		
Sex		0.038	0.846
Male	3.93 (0.83)		
Female	3.99 (0.71)		
Education		0.956	0.386
Associate’s degree	4.07 (0.74)		
Bachelor’s degree	3.97 (0.71)		
Master’s degree	3.78 (0.73)		
Hospital type		2.319	0.129
Tertiary hospital	4.02 ± 0.71		
Secondary hospital	3.85 ± 0.71		
Clinical experience		4.161	0.003
≤ 5	4.22 (0.68)		
6–10	4.12 (0.74)		
11–15	4.01 (0.64)		
16–20	3.74 (0.80)		
≥ 21	3.83 (0.64)		
Seniority		12.706	< 0.001
Primary title	4.29 (0.63)		
Intermediate title	3.82 (0.73)		
Senior title	3.90 (0.67)		
Frequency of truth telling		4.329	0.005
Seldom	3.77 (0.80)		
Sometimes	3.92 (0.60)		
Often	4.05 (0.73)		
Always	4.27 (0.78)

**Table 4:** Multiple regression models (*n* = 279)

Independent variables	B (95%CI)	SE	β	***p***
Constant	3.770 (3.418–4.122)	0.179		< 0.001
Seniority	−0.111(−0.167--0.055)	0.029	−0.222	< 0.001
Frequency of truth disclosure	0.162 (0.076–0.249)	0.044	0.212	< 0.001

*F* = 14.265, *p* < 0.001, adjusted *R*^*2*^ = 8.7%

### Nurses’ perception of difficulties in truth disclosure

The most common difficulty of truth disclosure was fear of patients’ negative emotions or their suicide attempts after truth disclosure, which was followed by families’ wish to conceal the truth, fear of getting into medical dispute due to truth disclosure, and lack of sufficient time for truth disclosure. Fourteen participants reported other difficulties such as fear of discordance with the physician’s truth disclosure and failure to understand patients’ conditions thoroughly (Table 5).

**Table 5:** Nurses’ perception of difficulties in truth disclosure (*n* = 279)

Item	***n***	%
Fear of patients’ negative emotions or their suicide attempts after truth disclosure	211	75.6
Families’ wish to conceal the truth	200	71.7
Fear of getting into medical dispute due to truth disclosure	130	46.6
Lack of enough time for truth disclosure	121	43.4
Fear of burdening self with telling “bad’ news.	101	36.2
Lack of appropriate room for truth disclosure	86	30.8
Lack of capacity to tell the truth	77	27.6
Other	14	5.0

## Discussion

Our research adds to the knowledge on collaboration in truth disclosure between nurses and physicians due to the paucity of evidence in the existing literature. In our study, 97.2% of the nurses had implemented truth disclosure in their daily work, which was much higher than in previous research [20]. There were many opportunities and situations when the nurse could provide information about patients’ condition, as nurses had the closest relationship with patients compared to other health care staff. However, truth telling was not a work that could be completed independently by nurses. Collaboration between physicians and nurses can make breaking bad news easier and accelerate the change in patients’ attitudes, perceptions, and beliefs [12]. Most of the nurses in this study responded positively to nurse–physician collaboration in truth disclosure, particularly in sharing patient information, which was consistent with previous research [28]. Improvements were needed to facilitate joint participation in truth telling and raise awareness of cooperation among nurses and physicians.

In addition, the study found that collaboration was influenced by nurses’ seniority and frequency of truth disclosure. Nurses with lower seniority and telling truth more frequently had better collaboration with physicians in truth disclosure. This finding was similar to that of a previous study. In that study, nurses who were younger and less experienced were also found to be more likely to cooperate with physicians [26]. This could be explained because they may feel less confident when informing the patients of their diagnosis or prognosis. Nurses with more experience in truth telling tended to know the importance of cooperating with physicians in truth disclosure [15]. Thus, they cooperated more with physicians.

In our study, we also investigated difficulties in telling the truth perceived by nurses. These findings were important because identifying potential difficulties could help find possible solutions to facilitate nurses’ participation in truth disclosure. In the present study, the five main difficulties of truth telling were partially the same as the barriers identified by physicians. In one study, the physicians listed five reasons for the concealment of cancer diagnoses, including lack of awareness of patients’ right to knowledge, cultural influences, insufficient medical resources and training, families’ financial concerns, and the need to protect physicians from violence [29]. In the present study, the most common barrier was fear of patients’ negative emotions or their suicide attempts after truth telling. This was similar to the results of another study [2]. Although breaking bad news is a distressing process for patients, it might potentially reduce patients’ psychological distress if the health care staff communicated with the patients in a patient-centered manner by considering their preferences [3]. The latest research also proved that patients being informed of the disease diagnosis had no impaired quality of life compared to those whose diagnosis was concealed from them [1, 30–32]. Nurses needed to be aware of this and assess patients’ and families’ willingness and preferred way to find out bad news.

The following two main barriers were culture-specific: families’ wish to conceal the truth, and fear of getting into medical dispute due to telling the truth. In mainland China, medical personnel take a family-centered approach to diagnoses/prognoses disclosure. It was the family who decided whether to tell the truth to patient. Medical personnel would follow the families’ decisions in order to avoid medical disputes [1]. Three other difficulties perceived by nursing staff were lack of time, room, and ability. In mainland China, nurses spend most of their time on drug treatment. Very limited time is spent on communicating patients’ condition. No separate room is used for communicating with patients, which also makes private conversations difficult to take place. Additionally, nurses tended to think that it was much better for patients’ chief physicians to tell the bad news because they had a better understanding of the patients’ disease and could answer patients’ questions more thoroughly. Nurses felt that they lacked the capacity to disclose because of their incomplete understanding of the patients’ conditions and scarce education about how to break bad news [33]. However, previous research has proved that nurses’ participation in truth disclosure accelerates the change in patients’ attitudes, perceptions, and beliefs [12]. It is necessary for nurse managers and educators to attach importance to the cultivation of nurses’ ability to tell truth in order to promote nurses’ role in this area [14]. Inter-professional training regarding diagnosis and prognosis-related communication are essential to empower nurses in truth-telling collaboration [21].

### Strengths and limitations

The current study aimed to investigate physician–nurse collaboration in truth disclosure. The findings helped us to recognize the current status of cooperation and found deficiencies. The findings served as a step in the quality improvement process and might provide information for clinical practice and future studies. The main limitation of our study was that it was conducted in a randomized manner. The vast majority of participants were women. The large discrepancies between the proportions of males and females may not facilitate a significant difference and affect the reliability of the results. In addition, although the Chinese version of the NPCS had sound reliability and validity, we highlighted that respondents should complete the questionnaire from the perspective of truth disclosure, and the possibility of response bias could not be excluded. Additionally, this study only assessed the degree of cooperation in truth disclosure from the nurse’s perspective, and future work is needed to evaluate physicians’ perceptions of collaboration because physicians and nurses hold different views [34].

### Implications

As collaboration has a positive effect on truth telling, it is of great importance to identify tailored interventions to promote physician–nurse collaboration. Based on the study results, we could conclude those potential efforts to remove difficulties and enhance nurses’ participation in truth telling, which may facilitate cooperation with physicians. Education on strengthening the role of nurses, raising nurses’ awareness of cooperation, and enhancing nurses’ ability may be useful [34]. Additionally, strategies used to build physicians’ respect and trust for nurses can also facilitate effective physician–nurse communication and improve nurses’ willingness to collaborate [28].

## Conclusion

Most of the nurses in this study responded positively to physician–nurse collaboration regarding truth disclosure, especially in the sharing of patient information. Nurses with lower seniority and telling truth more frequently had better collaboration with physicians. Further studies are required to test the potential interventions to promote cooperation between nurses and physicians in truth disclosure.

## Data Availability

The data analyzed for this manuscript are available from the corresponding author on reasonable request.

## Abbreviation

NPCS: Nurse–Physician Collaboration Scale
